# Esophageal emergencies: WSES guidelines

**DOI:** 10.1186/s13017-019-0245-2

**Published:** 2019-05-31

**Authors:** Mircea Chirica, Michael D. Kelly, Stefano Siboni, Alberto Aiolfi, Carlo Galdino Riva, Emanuele Asti, Davide Ferrari, Ari Leppäniemi, Richard P. G. ten Broek, Pierre Yves Brichon, Yoram Kluger, Gustavo Pereira Fraga, Gil Frey, Nelson Adami Andreollo, Federico Coccolini, Cristina Frattini, Ernest E. Moore, Osvaldo Chiara, Salomone Di Saverio, Massimo Sartelli, Dieter Weber, Luca Ansaloni, Walter Biffl, Helene Corte, Imtaz Wani, Gianluca Baiocchi, Pierre Cattan, Fausto Catena, Luigi Bonavina

**Affiliations:** 1 Department of Digestive Surgery, Centre Hospitalier Universitaire Grenoble Alpes, La Tronche, France; 2 Department of General Surgery, Albury Hospital, Albury, NSW 2640 Australia; 3 Division of General Surgery, IRCCS Policlinico San Donato, University of Milan Medical School, Milan, Italy; 4 Department of Emergency Surgery, University Hospital Meilahti Abdominal Center, Helsinki, Finland; 5 Department of Surgery, Radboud University Medical Center, Nijmegen, The Netherlands; 6 Department of Thoracic Surgery, Centre Hospitalier Universitaire Grenoble Alpes, La Tronche, France; 7 Department of General Surgery, Rambam Health Campus, Haifa, Israel; 8 Division of Trauma Surgery, School of Medical Sciences, University of Campinas, Campinas, SP Brazil; 9 General, Emergency and Trauma Surgery Department, Bufalini Hospital Cesena, Cesena, Italy; 10 Department of General Surgery, Università di Pavia, Pavia, Italy; 11 Shock Trauma Center at Denver Health, Denver, CO USA; 12 General Surgery and Trauma Team, University of Milano, ASST Niguarda Milano, Milan, Italy; 13 Cambridge Colorectal Unit, Cambridge University Hospitals, Addenbrooke’s Hospital, Cambridge, UK; 14 Department of Surgery, Macerata Hospital, Macerata, Italy; 15 Trauma and General Surgery, Royal Perth Hospital, Perth, Australia; 16 Division of Trauma and Acute Care Surgery, Scripps Memorial Hospital, La Jolla, CA USA; 17 Department of Surgery, Saint Louis Hospital, Paris, France; 18 Department of Surgery, Sheri-Kashmir Institute of Medical Sciences, Srinagar, India; 19 Department of Surgery, University of Brescia, Brescia, Italy; 20 Emergency Surgery Department, Parma University Hospital, Parma, Italy

**Keywords:** Esophageal perforation, Caustic ingestion, Emergency management, Foreign body ingestion, Esophageal trauma

## Abstract

The esophagus traverses three body compartments (neck, thorax, and abdomen) and is surrounded at each level by vital organs. Injuries to the esophagus may be classified as foreign body ingestion, caustic ingestion, esophageal perforation, and esophageal trauma. These lesions can be life-threatening either by digestive contamination of surrounding structures in case of esophageal wall breach or concomitant damage of surrounding organs. Early diagnosis and timely therapeutic intervention are the keys of successful management.

## Background

Injuries to the esophagus represent a rare but potentially lethal clinical condition. Emergency management is a challenge and mortality remains high. Timely and appropriate treatment of esophageal injuries (EI) is the most important determinant of patient outcomes. Management is multidisciplinary and involves emergency physicians, trauma, general and thoracic surgeons, anesthesiologists, otorhinolaryngologists, gastroenterologists, and radiologists. Due to the rarity of these injures, most clinicians will have limited personal experience with EI treatment. Therapy of EI is based on the location (neck, thorax, abdomen), the cause, and the extent of esophageal damage. A delay in providing appropriate treatment remains the dominant risk factor for mortality. Associated injuries of surrounding structures require specific treatment and may impact short-term survival.

The aim of the present review is to provide practitioners, who may be called upon to provide emergency management of EI, with a readily accessible comprehensive tool to help in the decision-making process.

## Methods

For the purpose of the paper, we used an etiological classification of esophageal injuries: (1) foreign body ingestion, (2) caustic ingestion, (3) esophageal perforations (iatrogenic and spontaneous), and (4) esophageal trauma. Leading specialists in the field were asked to perform a thorough MEDLINE and EMBASE search for relevant papers on each of these topics between 1985 and June 2018. They were asked to focus their search in order to provide evidence-based answers to pertinent questions with immediate practical application. Topics were presented and open to discussion at the 5th WSES congress in Bertinoro, Italy, 28th–30th June, 2018. The level of evidence for each recommendation statement was assigned by using the grading system proposed by the Oxford Centre for Evidence-Based Medicine [1].

Eventually, evidence-based guidelines for the management of EI were developed to outline clinical recommendations.

### Foreign body ingestion

In the USA, esophageal foreign body (FB) ingestion accounts for more than 100,000 cases per year. In children, accidental ingestion of coins, batteries, toys, and magnets is common. Accidental ingestions also occur in adults often in association with intoxication or in the elderly with cognitive impairment; intentional ingestion by patients with psychiatric disorders or by prisoners is not infrequent [2–4]. Esophageal FB impaction depends on the size and shape of the FB. Impaction usually occurs at the level of the hypopharynx or in the upper thoracic esophagus for anatomical (cricopharyngeus, aortic arch) and physiological reasons (low pressure zone at the transition point between striated and smooth muscle fibers) [5, 6]. Non-impaired adults and older children can typically identify foreign body ingestion and may point to a specific area of discomfort. However, children and mentally impaired adults may not give a history of foreign body ingestion [2]. The typical clinical presentation is the acute onset of dysphagia or inability to swallow saliva. Other related clinical features are odynophagia, neck tenderness, retrosternal pain, sore throat, foreign body sensation, retching, vomiting, and drooling. Choking, stridor, and dyspnea may be present in patients with airway obstruction or aspiration. Physical examination findings include the presence of fever, cervical subcutaneous emphysema or erythema and tenderness in the event of complications [6–8].

#### Which are the appropriate biochemical and imaging investigations?

Initial evaluation should be based on the patient’s history and physical examination. Recommended biochemical investigations are complete blood count (CBC), C-reactive protein (CRP), blood gas analysis for base excess, and lactate (Grade 2C).

Neck, chest, and abdominal radiographs are useful to assess the presence, location, shape, and size of radiopaque or unknown shape objects (Grade 1C). Plain neck, chest, and abdominal radiographs are useful to assess the presence, location, size, shape, and number of ingested objects and possible signs of perforation. Plain radiography is usually employed for the initial screening but the false-negative rate is up to 47%. Biplanar radiography is useful to reduce the false-negative rate and the lateral projection is important to differentiate between tracheobronchial and esophageal FBs. In case of food bolus impaction, thin metal objects, wood and plastic objects, glass fragments, fish or chicken bones, false-negative rates at the X-ray evaluation are up to 85% [9, 10].

Computed tomography (CT) scan should be performed in patients with suspected perforation or other complications that may require interventional endoscopy or surgery (Grade 1B). In a prospective single-center study including 358 adult patients with symptomatic fish bones impaction the sensitivity of plain X-Ray was 32% while the sensitivity of CT scan was 90–100% and the specificity 93.7–100%. For this reason, CT scan should be considered an essential tool in adult patients reporting accidental ingestion or suspected ingestion of bone fragments and negative X-rays. In addition, CT scan is necessary if there is suspicion of FB-related complication (perforation, abscess, mediastinitis, aortic/tracheal fistulas) [11–13].

Contrast swallow is not recommended and should not delay other investigations/interventions (Grade 1B). Oral contrast studies (barium or gastrografin studies) should be avoided in patients with complete esophageal obstruction and inability to swallow saliva because of the increased risk of aspiration. In addition, barium swallow may coat the foreign body and esophageal mucosa impairing endoscopic visualization. In any case, oral contrast studies should not delay other investigations/interventions [14, 15].

#### What are the indications for endoscopy?

Therapeutic flexible endoscopy is recommended as first-line treatment of persistent esophageal foreign bodies (Grade 1B), although 80–90% of ingested foreign bodies pass spontaneously through the gastrointestinal tract. In patients with persistent esophageal symptoms, an endoscopic evaluation should be performed, even if the radiographic examination is negative. In addition, in patients with food bolus impaction and no evidence of complications, endoscopy may be performed first [16–18]. It will depend on local practices but most cases will require anesthetic input and often a general anesthetic with endotracheal intubation will be used to protect the airway.

**Fig. 1:**
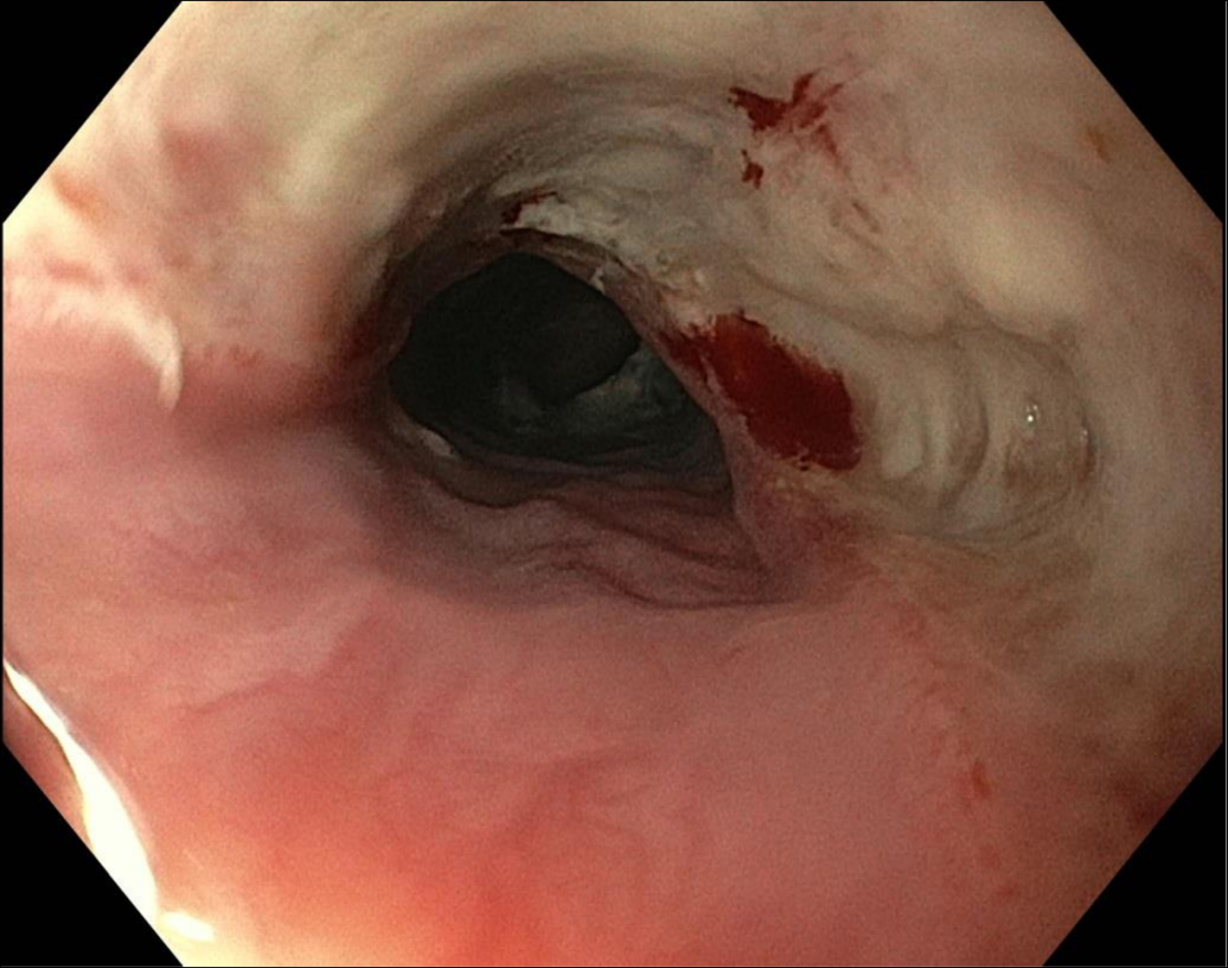
Endoscopic view of esophageal injury from button battery ingestion (at 6 h) in a 5-year old with intellectual disability

Emergent flexible endoscopy (preferably within 2 h, at latest within 6 h) is recommended for sharp-pointed objects, batteries, magnets, and for foreign bodies inducing complete esophageal obstruction (Grade 1B). Emergent flexible endoscopy should be performed (a) in case of sharp-pointed objects because of the high risk of full-thickness perforation (up to 35%); (b) in case of button/disk battery ingestion because of the risk of pressure necrosis, electrical burns, and chemical injury (Fig. 1); (c) in case of magnet ingestion due to pressure necrosis; and (d) in case of food bolus ingestion with complete esophageal obstruction because of the risk of aspiration as well as perforation [19–22].

Urgent (< 24 h) flexible endoscopy is recommended for other esophageal foreign bodies without complete obstruction (Grade 1B) [19–22].

Gently pushing the bolus into the stomach is recommended for the treatment of esophageal food bolus impaction. If this procedure is not successful, retrieval should be considered (Grade 1C). It has been shown that in case of food bolus impaction, air insufflation and gentle instrumental pushing (push technique) is associated with a low complication rate and up to 90% success rate. If a large FB is jammed in the lower esophagus, push technique may impact it further; gentle passage of a balloon catheter (ERCP stone extraction catheter) past the FB and inflation of the balloon with withdrawal can be used to try to disimpact the FB which may then be retrieved in a net. Retrieval techniques using baskets, snares, and grasping forceps should be considered in case of resistant or sharp-pointed objects [18, 23]. A combination of techniques may be required in difficult cases.

In addition to therapeutic endoscopy, diagnostic work-up for potential underlying disease including histological evaluation is recommended (Grade 1B). An underlying esophageal disorder can be found in up to 25% of patients. The most commonly associated disorders are esophageal stricture, hiatus hernia, esophageal web or Schatzki ring, eosinophilic esophagitis, achalasia, and tumors. A latent eosinophilic esophagitis may be diagnosed in up to 9% of patients [6, 8, 24–26].

Flexible and rigid endoscopy are complementary/cross-over techniques. Flexible endoscopy remains the “first line” approach to FB; rigid endoscopy has a place as a “second line” therapy (Grade 2B). Rigid endoscopy through rigid endoscopes, should be considered in case of FB located in the upper esophagus (Achilles’ heel of flexible endoscopy) and in case of FB ingestion with concomitant respiratory symptoms or suspicion of FB in the upper airways [26–28]. The use of the bivalved Weerda diverticuloscope is another option as it allows dilation and opening of the upper esophageal sphincter. A combined approach using a flexible endoscope introduced through the Weerda diverticuloscope is also feasible [29, 30]. In addition, through the diverticuloscope, it is possible to use laparoscopic grasping forceps for retrieval. A recent meta-analysis comparing flexible versus rigid endoscopy for retrieval of upper esophageal FB showed that both were effective and safe, with similar success and overall complication rates [31].

#### Who should undergo surgical treatment and what is the appropriate timing for surgery?

Potential indications for surgical treatment include irretrievable foreign body, perforation, FB close to vital structures (aortic arch), and other complications (Grade 1B). Upfront surgery should be adopted immediately in case of esophageal perforation with extensive pleural/mediastinal contamination (Grade 1B). Up to 1–3% of patients require surgery because of complications (perforation, irretrievable foreign bodies, mediastinitis, pleural empyema, fistula, severe bleeding) [5, 6, 15, 18, 32].

#### What are the most appropriate surgical procedures?

The surgical approach depends on the location of FB impaction, patient comorbidities, and patient condition (Grade 1B). Minimally invasive techniques should be considered first-line treatment in referral centers (Grade 1C). Esophagotomy with FB extraction and primary closure should be considered in case of limited pleural/mediastinal contamination and vital edges (Grade 1C). Different surgical approaches may be used according to FB location and patient comorbidities (left cervicotomy, right/left thoracotomy, minimally invasive right/left thoracoscopy, prone thoracoscopy, laparoscopy, and laparotomy) [5, 6, 15]. Open or minimally invasive esophagotomy with primary repair can be used in cases of limited mediastinal contamination and vital edges of the perforation. Rescue esophagectomy with primary or delayed reconstruction should be considered in case of extensive contamination [33–37] .

### Corrosive ingestion

Corrosive ingestion is a rare but potentially devastating event that can result in patient death. In survivors, it is responsible for swallowing troubles, impaired quality of life, and significant burdens on health systems. The real incidence is currently unknown as the ingestion of corrosive agents is probably largely underreported around the world [38, 39]. In children, ingestion is mostly accidental and severe injuries are rare. Massive suicidal ingestion of strong corrosive agents occurs usually in adults suffering psychiatric disease and requires aggressive emergency management. It is commonly accepted that clinical symptoms do not correlate reliably with the extent of gastrointestinal damage; the absence of pain and of oral lesions does not rule out life-threatening gastrointestinal injuries [38–44]. Appropriate management of corrosive injuries in the emergency setting affects patients’ outcomes [45].

#### What are the possible etiologies and how do they affect the clinical presentation and the therapeutic options?

Strong acids and alkalis are responsible for most severe caustic injuries to the gastrointestinal tract. Identification of the nature, the physical form, and the quantity of the ingested agent as well as the accidental-voluntary ingestion pattern are the cornerstones for emergency management of corrosive injuries (Grade 2A). Contacting Poison Control Centers to evaluate systemic toxicity of the ingested agents is recommended (Grade 2B). In case of massive ingestion, both acids and alkalis may induce extensive necrosis of the gastrointestinal tract [45]. Oxidants (bleach) usually cause mild injuries but severe damage requiring emergency resection has been occasionally reported [45]. Ingestion of ammonia results in superficial hemorrhagic gastritis which may progress during the first 24–48 h and requires specific surveillance [38]. The quantity of ingested corrosive agent is related to the accidental/voluntary pattern of ingestion; this is the most important prognostic factor although reliable information is usually lacking [46]. The physical form of the ingested substance is another major determinant of the damage pattern to the gastrointestinal tract. Solids produce maximum damage to the mouth and the pharynx, while liquids transit rapidly and induce burns of the esophagus and the stomach; concomitant vapor aspiration (ammonia, formaldehyde) may cause airway burns. Caregivers should be aware that specific corrosives may also cause severe systemic effects such as hypocalcemia (phosphoric, hydrofluoric acids), hyponatremia (strong acids/alkalis), hypokalemia, and acidosis [38].

#### What are the appropriate biochemical and imaging investigations?

Initial laboratory evaluation of caustic injuries should include CBC, serum concentrations of sodium, potassium, chlorine, magnesium, calcium, urea creatinine, liver tests (bilirubin, alanine aminotransferase, aspartate aminotransferase), pH and serum lactate, blood alcohol levels, and measurement of β-HCG in young women (Grade 2A). Laboratory and imaging findings have an important role in identifying patients with transmural necrosis who might benefit from emergency surgical treatment. As initial normal laboratory values do not rule out transmural necrosis, kinetics of laboratory data is useful in patient monitoring and management [47, 48]. Abnormal values such as severe acidosis (low pH, high blood lactate levels) [49], deranged liver function tests [49], leukocytosis, elevated CRP level [39], renal failure [47], and thrombocytopenia [50] are predictive of transmural necrosis and poor outcomes.

**Fig. 2:**
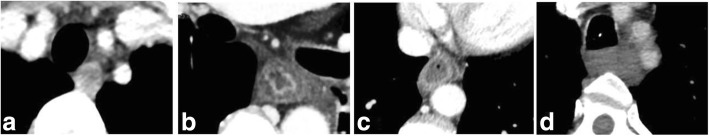
CT classification of corrosive injuries of the esophagus. **a** Grade I—homogenous enhancement of the esophageal wall while wall edema and mediastinal fat stranding are absent. **b** Grade IIa—internal enhancement of the esophageal mucosa and hypodense aspect of the esophageal wall which appears thickened, concomitant enhancement of the outer wall confers a “target” aspect. **c** Grade IIb—fine rim of external wall enhancement, the necrotic mucosa does not enhance anymore and fills the esophageal lumen which shows liquid density. **d** Grade III injuries show the absence of post-contrast wall enhancement

Neck, chest, and abdominal radiographs may show the presence of free air in patients with gastrointestinal perforation (Grade 3A). Emergency management of caustic ingestion can be performed safely relying on computed tomographic evaluation (Grade 2A). Recent studies have shown that emergency contrast-enhanced computed tomography (CT) examination outperformed endoscopy in detecting transmural injuries of the gastrointestinal tract after caustic ingestion and in predicting esophageal stricture formation [48, 51, 52]. CT of the neck, the thorax, and the abdomen should be performed 3–6 h after ingestion, before and after intravenous injection (2–3 mL/s) of a nonionic contrast agent (Iomeron 350; 2 mL/kg), with 18- to 25-s acquisition time and a 90-s scan delay. The main sign of transmural digestive necrosis is the absence of post-contrast wall enhancement, and its presence at any level (esophagus, stomach, duodenum, bowel, colon) is an indication for emergency surgery [38]. A four-stage CT classification of esophageal caustic injuries (Fig. 2) can be used in which: *Grade I* injuries show homogenous enhancement of the esophageal wall while wall edema and mediastinal fat stranding are absent; *Grade IIa* injuries display internal enhancement of the esophageal mucosa and hypodense aspect of the esophageal wall which appears thickened while concomitant enhancement of the outer esophageal wall may sometimes confer a “target” aspect; *Grade IIb* injuries present as a fine rim of external wall enhancement; the necrotic mucosa does not enhance and fills the esophageal lumen which shows liquid density. Mediastinal fat stranding is uniformly present in Grade II esophageal injuries. *Grade III* injuries show the absence of post-contrast wall enhancement.

#### What is the role of endoscopy and endoscopic treatment?

Emergency endoscopy should be performed if (1) CT is unavailable, (2) CT with contrast administration is contraindicated (renal failure, iodine allergy, etc.), (3) CT suggests transmural esophageal necrosis but interpretation is difficult/uncertain, or (4) in the pediatric population (Grade 2A). Endoscopy used to be the mainstay of management algorithms following caustic ingestion [45, 53]. The major drawback of endoscopy is its inability to predict accurately transmural necrosis, which may expose patients to either futile surgery or inappropriate “watch and wait” management and risk of death. The use of a CT-based algorithm to select patients for emergency surgery significantly improved patient outcomes when compared to endoscopy-based management [48, 51, 54]. The role of emergency endoscopy evaluation of caustic injuries is currently reduced to situations in which CT cannot be employed. Endoscopy remains the upfront evaluation examination in children as severe injuries are rare and long-term effects of radiation exposure are an important issue [38]. The Zargar endoscopic classification [54] of caustic injuries is most commonly employed; its ability to predict stricture formation remains controversial [55] and is outperformed by CT [52].

Endoscopy is the main diagnostic tool of esophageal/gastric strictures in symptomatic patients (Grade 2A). Stricture formation is the most common and disabling long-term complication of corrosive ingestion. Strictures more frequently involve the esophagus than the stomach and usually occur within 4 months after ingestion [52, 53]. Dysphagia and regurgitation are the main symptoms of corrosive strictures and should prompt immediate upper gastrointestinal evaluation [56].

Endoscopic dilation is the upfront treatment of esophageal strictures. Endoscopic dilation should be attempted 3–6 weeks after ingestion in patients with few (< 3) short (< 5 cm) esophageal strictures (Grade 2A). Reconstructive esophageal surgery should be considered after recurrent failure of endoscopic dilation (Grade 2A). Corrosive strictures can involve all esophageal segments; are often multiple, long, irregular; and have long stabilization delays [57]. Endoscopic dilation is the first-line management option [39]. Dilation can be started safely after healing of acute injuries, usually between the 3rd and the 6th week and the interval between dilations varies between 1 and 3 weeks. Three to 5 sessions are expected to provide satisfactory results [39], and esophageal reconstruction should be considered after 5–7 failed attempts [58]. The advent of interventional endoscopy has renewed the interest of intraluminal stenting, but solid data supporting this approach is still lacking.

#### What are the indications for non-operative management?

Patients who do not have full-thickness necrosis of digestive organs should undergo non-operative management (Grade 1C). Patients eligible for non-operative treatment require close clinical and biological monitoring. Any deterioration in the condition of the patient should prompt repeat CT examination and consideration for surgery (Grade 2A). Oral feeding should be reintroduced as soon as patients swallow normally. Enteral feeding by nasogastric tubes or jejunostomy construction is recommended in patients unable to eat. Psychiatric evaluation is mandatory in all patients prior to hospital discharge (Grade 2C). Patients who do not show signs of transmural necrosis of the gastrointestinal tract on emergency CT are eligible for non-operative management [48, 51]. Subsequent deterioration in clinical symptoms and signs (rebound tenderness, increasing abdominal pain, shock, need for ventilator support, etc.) or of laboratory tests (renal failure, acidosis, leukocytosis, etc.) suggest evolution of injuries to transmural necrosis (5% of patients) and should prompt repeat CT evaluation [38]. Patients with *Grade I* CT injuries can be fed immediately and discharged quickly (24–48 h) from the hospital. Long-term follow-up is not required in these patients as the stricture formation risk is nil. Patients with *Grade IIa* CT esophageal injuries have a low risk (< 20%) of stricture formation [52]. Oral nutrition is usually well tolerated and should be introduced as soon as pain diminishes and patients can swallow. Patients with *Grade IIb* CT esophageal injuries are at high risk (> 80%) of stricture formation [52]. Pain during deglutition, hyper-salivation, and early dysphagia may hinder early oral intake; if symptoms persist, nutritional support by long-term parenteral nutrition or feeding jejunostomy is required. A 4–6 months post-ingestion visit is recommended for patients with *Grade II* CT injuries as most strictures develop within this delay. Psychiatric evaluation is mandatory in all patients prior to hospital discharge; long-term control of the psychiatric disease is important to avoid recurrence [38].

#### What are the indications for surgical treatment?

Surgery should be performed as soon as possible in patients with caustic necrosis to avoid death (Grade 1C). All obvious transmural necrotic injuries should be resected during the initial operation (Grade 2A). A feeding jejunostomy is indicated at the end of the operation (Grade 2A). Emergency surgery is indicated if the initial evaluation suggests transmural necrosis of the gastrointestinal tract (Grade III CT injuries) [38]. In the absence of appropriate management, necrosis of intraabdominal organs eventually leads to perforation, peritonitis/mediastinitis, and death [59, 60]. The decision to perform an emergency operation after corrosive ingestion is a life-changing event for the patient; in a recent report, the standard mortality ratio of patients operated for caustic necrosis was 21.5 when compared to the general population [45]. Laparotomy remains the standard approach in the emergency setting although successful laparoscopic management has been reported [61, 62]. All obvious transmural necrotic injuries should be resected during the initial procedure; reoperation should be undertaken promptly if ongoing necrosis is suspected [63]. Stripping esophagectomy and gastrectomy, performed through a combined abdominal and cervical approach is indicated in patients with transmural necrosis of both the esophagus and the stomach [45, 59, 60]. Esophageal reconstruction should be prohibited at the time of the emergency procedure because subsequent stricture formation can compromise functional outcomes. If necrosis is confined to the stomach, total gastrectomy with preservation of the native esophagus or esophageal diversion should be considered [38]. Immediate esophagojejunostomy reconstruction can be performed safely with low leak rates (5–8%) [64]. Partial gastric resections are not recommended because ongoing necrosis might compromise patient survival. Isolated esophageal necrosis justifying esophagectomy with gastric preservation has been recently challenged [47, 50]; non-operative management may be attempted in these patients in the absence of transmural gastric necrosis. Concomitant necrosis of adjacent organs (spleen, colon, bowel, duodenum, and pancreas) requires extended resections at the time of esophagogastrectomy in up to 20% of patients [45, 63]. If pancreatoduodenectomy is undertaken for corrosive injuries, immediate pancreato-biliary reconstruction is recommended [65]. Preoperative tracheobronchial endoscopy is mandatory to detect tracheobronchial necrosis resulting from mediastinal extension of esophageal necrosis; in this situation pulmonary patch repair through a right thoracotomy approach may be lifesaving [66]. Resection should be abandoned if extensive bowel necrosis is found at laparotomy because of poor survival and compromised nutritional issues [63].

### Esophageal perforations

Esophageal perforation (EP) covers a large range of conditions characterized by the transmural disruption of the esophagus [67]. Spontaneous esophageal perforation (Boerhaave syndrome) is most often due to an abrupt increase in the esophageal pressure following a vomiting effort in the absence of relaxation of the superior esophageal sphincter. It accounts for 15% of esophageal perforations; the tear is usually located on the left border of the lower third of the thoracic esophagus and the wall defect is large (3–8 cm) [68–70]. The large majority (60%) of esophageal perforations are iatrogenic and occur during diagnostic and therapeutic (esophageal dilation, varices ligation, sclerotherapy, etc.) endoscopic procedures [71]. Other rare causes include operative and external trauma, malignancy, foreign bodies, and caustic ingestion. Forceful retching or vomiting causing perforation has erroneously come to be known as spontaneous esophageal perforation; as it is not spontaneous it may be better to use other terms such as barogenic rupture or Boerhaave syndrome [72].

The common denominator of all these heterogeneous conditions is the contamination of surrounding spaces with digestive contents and the evolution to severe sepsis and death in the absence of timely diagnosis and appropriate treatment. Mortality of esophageal perforation ranges between 10% and 20% and the delay in treatment is the most important survival predictor [73, 74].

#### What are the appropriate laboratory and imaging studies?

Routine blood tests (CBC, serum concentrations of sodium, potassium, chlorine, magnesium, calcium, urea creatinine, liver tests (bilirubin, alanine aminotransferase, aspartate aminotransferase), pH and serum lactate) should be performed in patients with suspected EP (Grade 1C). The initial clinical and biological presentation of EP has no specific patterns; late stages are characterized by signs of inflammation and sepsis. To avoid delay in diagnosis (> 50% of cases) and allow timely management, a high degree of suspicion is required at presentation [68, 75, 76].

Contrast-enhanced computed tomography (CT) and CT esophagography is the imaging examination of choice in patients with suspicion of EP (Grade 1C). CT is highly sensitive (92–100%) in detecting EP and helps to asses extension to adjacent structures (collection of air or fluid in the mediastinum, pleural and intra-peritoneal effusions) and to guide initial therapy. CT can also eliminate other conditions that may mimic EP (aortic dissection, esophageal intramural hematoma, etc.) [13, 67, 77, 78]. In select cases, contrast-enhanced esophagogram (gastrografin/barium) may provide useful information regarding the location and the contained character of EP [78]. Indirect signs of esophageal injury can also be seen on a plain chest radiograph (pleural effusion, pneumomediastinum, subcutaneous emphysema, hydrothorax, pneumothorax, and collapse of the lung) [79].

#### What is the role of endoscopy and endoscopic treatment?

Diagnostic endoscopy is useful in patients with suspected EP and doubtful CT findings. (Grade 1C). Diagnostic endoscopy for EP is reliable and safe in experienced hands; nevertheless, potential risks of enlarging the perforation size and aggravating the contamination of surrounding spaces warrant caution and limit its use as a first-line exam [71].

Endoscopic treatment is the gold standard for closing EP that occur and are recognized during an endoscopic procedure (Grade 2A). New interventional endoscopic techniques, including endoscopic clips, covered metal stents, and endoluminal vacuum therapy, have been developed over the last several years to manage esophageal perforation in an attempt to decrease the related morbidity and mortality [80]. Endoscopic clip placement (through the scope clips, over the scope clips) is currently the standard method for closing small (< 2 cm) luminal perforations [81–83]. Endoscopic stents (partially or fully covered self-expandable metal stents, self-expandable plastic stents) can be used to cover larger defects or complete unsatisfactory clip closure [84]. In a recent review, the use of self-expandable stents for the treatment of esophageal leaks (spontaneous, iatrogenic, and postoperative) resulted in 88% success and 7.5% mortality rates. These results compared favorably with outcomes of surgery (83% success and 17% in hospital mortality) leading the authors to conclude that esophageal stenting can be successfully applied as an alternative therapeutic strategy in EP [85]. Minimal 2–4-week duration of stent placement has been advocated to allow sealing of the perforation. Esophageal stent placement is probably just as effective as surgical repair for the treatment of iatrogenic EP [86]. Endoscopy may be used as definitive treatment either alone or in combination with interventional radiology or surgical procedures (drainage of pleural abscess, or compressive pneumothorax, etc.) [71]. Successful closure of esophageal defects by primary or rescue endoluminal vacuum therapy has been recently reported and may represent a promising alternative treatment for EP [87, 88].

In patients with late presentation and in patients with non-endoscopic EP, the use of endoscopy as first-line therapy may be considered (Grade 2C). Although successful endoscopic management has been reported in select Boerhaave [89–91] patients with minimal symptoms and signs of sepsis, concerns on patient safety warrant caution regarding first-line use of endoscopic treatment under such circumstances [71, 89]. Endoscopic stenting is a useful adjunct treatment tool in patients with persistent leakage following surgical treatment of EP [92].

#### What are the indications for non-operative treatment?

Non-operative management (NOM) of EP can be considered in stable patients with early presentation, contained esophageal disruption, and minimal contamination of surrounding spaces if highly specialized surveillance is available (Grade 1C). The criteria developed by Altorjay et al. [93] more than two decades ago are still the mainstay of non-operative management (Table 1). More recently, the Pittsburgh classification has been developed to include an esophageal perforation score based on ten clinical and radiological factors to help decision-making for patients with EP [94]. The score has been validated in a multinational study, and it has been suggested that low score (≤ 2) patients might be eligible for non-operative management [95].

**Table 1:** Criteria for non-operative management of esophageal perforations

Delay in management	Early: less than 24 h
Clinical presentation	Absence of symptoms and signs of sepsis
Radiological criteria	Cervical or thoracic location of the esophageal perforation Contained perforation by surrounding tissues - Intramural - Minimal peri-esophageal extravasation of contrast material with intra-esophageal drainage - Absence of massive pleural contamination
Esophageal characteristics	No preexistent esophageal disease
Other	Possibility of close surveillance by expert esophageal team Availability of round the clock surgical and radiological skills

Patients eligible for NOM should be kept on *nil* per os, administered broad spectrum antibiotics (aerobic and anaerobic bacteria), and proton pump inhibitor therapy (Grade 1C). Early introduction of nutritional support by enteral feeding or total parenteral nutrition is essential for esophageal healing (Grade 1C). Endoscopic placement of a nasogastric tube is recommended (Grade 2A). Although anti-infective treatment is considered a cornerstone in the management of EP, there is a lack of consensus regarding the optimal antibiotic regimen and the treatment duration. A recent review of the literature revealed the need for high-quality evidence related to anti-infective treatment in patients diagnosed with EP [96]. Additional measures should target sepsis control by using percutaneous radiology techniques to drain peri-esophageal and pleural collections [97]. Drainage of pleural collections and pleural decortication by video-thoracoscopy and use of endoscopic techniques (clips, stents, and internal vacuum drainage) are part of an aggressive minimally invasive management of EP. By using such a combined strategy Vogel et al. were able to perform successful NOM in 68% of 47 EP patients with a low mortality rate (6%) [98].

#### What are the indications for surgery?

Surgery should be undertaken in all patients who do not meet NOM criteria (Grade 1C).

If surgery is indicated for EP, patients should be taken to the operative room as soon as possible (Grade 1C). Even minor delays in surgical treatment may increase morbidity and mortality rates. Mortality of patients managed within 24 h of EP is under 10% compared to 30% after this time [68, 76, 78, 94].

Repair of EP by a minimally invasive surgical (laparoscopy, thoracoscopy) approach may be considered (Grade 1C). Reports are scarce and such an approach should probably be reserved to centers in which highly specialized expertise is available [99, 100].

#### What are the most appropriate surgical procedures?

General principles of esophageal perforation management include (1) excellent exposure, (2) debridement of non-viable tissue, (3) closure of defect, (4) use of buttress to reinforce esophageal sutures, and (5) adequate tube drainage. The surgical approach should be tailored according to the location of EP.

##### Cervical EP

For EP located in the neck, direct repair of the esophageal defect should be attempted whenever feasible (Grade 1C). The esophagus is approached through a left neck incision along the anterior border of the sternocleidomastoid muscle or by a collar incision if bilateral cervical exploration is required [74, 78]. Surgical treatment includes circumferential esophageal mobilization to facilitate repair, debridement of the perforation site, single- or double-layer tension-free closure of the perforation, buttressing of the repair with vascularized tissue (sternocleidomastoid muscle, digastric muscle), and adequate drainage [74]. Placement of a feeding tube (nasogastric, jejunostomy) at the time of repair allows early nutritional support and favors healing [68].

If direct repair is not feasible (disruption exceeds 50% of the esophageal circumference, delayed surgical exploration), external drainage is recommended (Grade 2A). Construction of a lateral or end esophageal stoma should be considered to decrease contamination of surrounding spaces.

##### Thoracic EP

Primary repair is the treatment of choice for EP with free perforation of the thoracic esophagus (Grade 1C). Management of perforation of the thoracic esophagus relies on immediate interruption of mediastinal and pleural contamination, debridement of the perforation to healthy tissue, tension-free primary repair, and adequate external drainage [101].

**Fig. 3:**
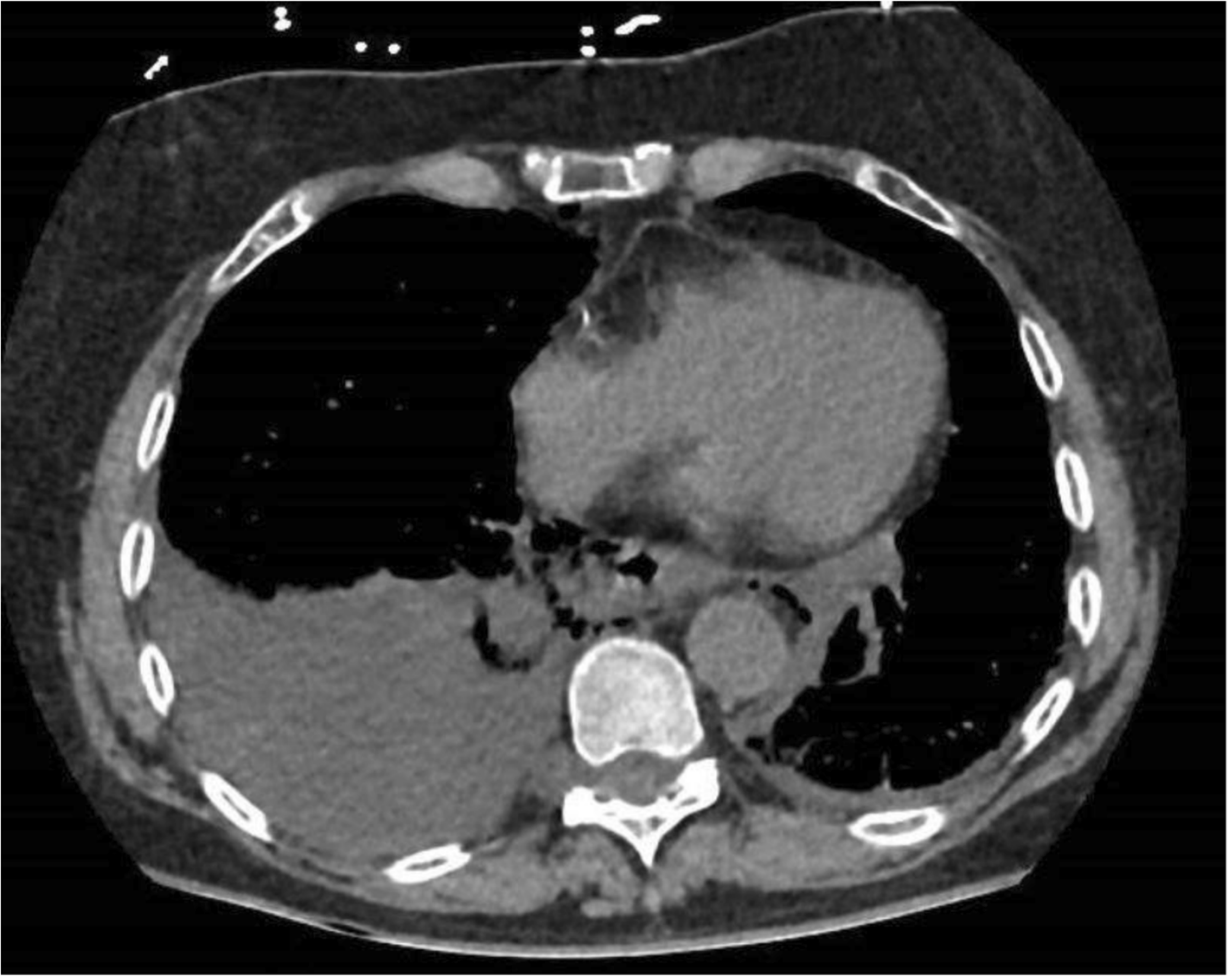
Axial CT showing a right pleural effusion, mediastinal air and esophageal wall disruption in a patient with spontaneous EP (Boerhaaves). Patient managed by right thoracotomy and laparotomy

**Fig. 4:**
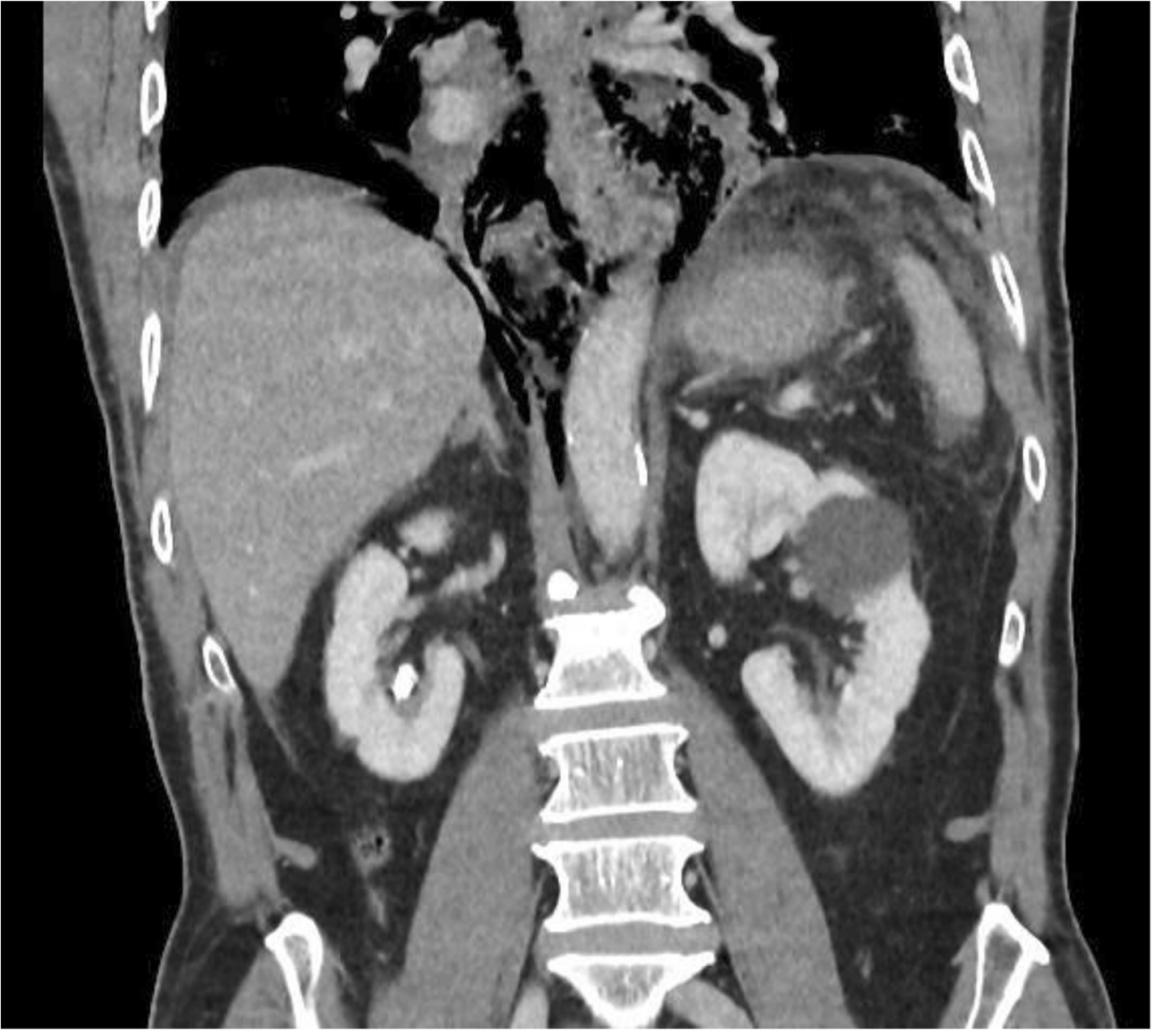
Coronal CT showing mediastinal air but minimal pleural reaction in a patient with spontaneous EP (Boerhaaves). The patient was successfully managed via laparotomy alone and transhiatal repair. Primary suture repair with interrupted full-thickness single-layer polyglycolic acid and fundoplication healed without a leak

These cases demand an individualized approach and it is difficult to be proscriptive about the actual operative steps. Thoracotomy will usually be required and the degree of pleural effusion or visible wall defect on CT may guide the incision side (Fig. 3). A laparotomy or laparoscopy will usually be required in addition to enable construction of a feeding jejunostomy and possibly a decompressive tube gastrostomy. The alternative is a nasogastric tube or combination of tubes to allow decompression and feeding. In general, a diversionary cervical esophagostomy (for saliva) is not recommended. In some patients with suitable body habitus, a transhiatal approach via a midline laparotomy may be used (Fig. 4). Excision of the xiphoid coupled with use of a sternal hook retractor can allow repair of thoracic esophageal perforations without thoracotomy. The fundus will need to be mobilized and the esophagus encircled with a tape to allow full mobilization and dissection high up into the mediastinum.

The mucosal defect is often longer than the muscular tear; longitudinal myotomy at both ends of the EP is useful to expose mucosal edges for appropriate repair [68]. Two-layer repair, with separate suturing of the mucosa and muscle has traditionally been recommended. The risk of suture breakdown is generally quoted to be between 25 and 50%. Buttressing the esophageal repair with surrounding viable tissue (intercostal muscle flap, pleural or pericardic patch) has been recommended to decrease the risk of leakage. In cases approached transhiatally, a Nissen fundoplication can be an effective buttress of the repair. Drainage of the mediastinum and pleural cavity is required and enteral nutrition remains an essential component of the treatment plan.

If direct repair of thoracic EP is not feasible (hemodynamic instability, delayed surgical exploration, extensive esophageal damage) esophageal exclusion, diversion, or resection should be performed (Grade 1C). Repair over a large size T-tube can be used to create a controlled esophago-cutaneous fistula and minimize mediastinal and pleural contamination [102]. Complete esophageal diversion or thoracic esophageal resection is required in the presence of large esophageal disruption; creation of a cervical esophagostomy and feeding jejunostomy are mandatory in these patients [101]. Resection is the best option in the presence of pre-existing esophageal pathology [68, 103]. If the patient survives, colon interposition or gastric pull-up reconstruction are required 6–12 months after complete diversion or resection of the thoracic esophagus.

##### Abdominal EP

Operative repair is the treatment of choice for patients with free perforation of the abdominal esophagus (Grade 1C). Abdominal esophageal perforation should be approached by a midline laparotomy. Following debridement of necrotic tissues, single- or double-layer tension-free closure of the perforation should be performed. It is recommended to buttress the esophageal suture with a gastroplasty using the gastric fundus (i.e., complete or partial fundoplication), position a nasogastric tube, construct a feeding jejunostomy, and perform external drainage of the subphrenic space [78].

### Esophageal trauma

Injury of the esophagus by external trauma is a rare condition. Traumatic injuries of the esophagus (TIE) account for less than 15% of all esophageal injuries [104, 105]. TIE were recorded in less than 1% of patients managed in 20 Level I trauma centers across a 6-year period [106]. They are classified according to the anatomic location, i.e., cervical, thoracic, or abdominal and according to the mechanism of injury, i.e., penetrating and blunt trauma. An unusual cause of TIE is barotrauma by external air-blast injuries [107]. Due to the anatomical situation of the esophagus, isolated TIE are rare; associated injuries to the spinal cord, airway, major vascular structures, lungs, heart, and abdominal viscera (spleen, pancreas, liver) are common and worsen the prognosis [108, 109]. TIE occurs mostly in young males and the most frequently encountered presentation is that of a penetrating injury to the cervical esophagus. Mortality of TIE is high with most deaths occurring within 24 h because of severe associated injuries [105]. Trauma to the thoracic esophagus is especially associated with high mortality rates [110]. Early diagnosis of TIE is mandatory to improve outcomes and requires a high level of suspicion.

#### What is the appropriate diagnostic work-up?

Physical examination is not reliable for early diagnosis of TIE (Grade 2A). There are no specific symptoms or pathognomonic signs of TIE. Pointers to TIE include thoracic pain (70%), fever (50%), dyspnea (25%), subcutaneous emphysema (19%), and dysphagia (7%).The mechanism of injury outperforms clinical signs in establishing early diagnosis of TIE [109, 111].

Laboratory studies are not useful for early diagnosis of TIE (Grade 2A). Biological modifications such as leukocytosis, increased CRP, and increased procalcitonin are non-specific and are related to the inflammatory response. Similarly, the presence of lactic acidosis, anemia, and coagulopathy are related to shock rather than TIE [104].

Contrast-enhanced CT and CT esophagography should be performed in hemodynamically stable patients with suspicion of TIE (Grade 1C). CT esophagogram has high sensitivity (95%) and specificity (91%) rates in detecting upper digestive tract perforation. Contrast-enhanced CT is useful to identify associated injuries and can provide important information regarding the trajectory of the penetrating agent (bullet, stab wound). CT may also show indirect signs of esophageal perforation (paraesophageal collections, free air, pleural effusions). Over the past years, CT has largely replaced contrast (gastrografin/barium) esophagogram, which was the test of choice for years but provides less information, requires a stable and cooperative patient, and can miss up to 30% of small esophageal perforations [112, 113]. One major drawback of esophageal opacification techniques is the fact that swallowing is only possible in patients who are well; nasogastric tube-administered contrast may miss esophageal perforation.

#### What is the role of diagnostic endoscopy?

Flexible endoscopy should be performed as an adjunct to CT in patients with suspected TIE (Grade 2A). Endoscopy provides direct visualization of the injury site and was shown to be useful in patients with equivocal CT findings. Other advantages include easy availability in most trauma centers and the possibility of use in intubated and unstable patients [114, 115]. In combination with contrast-enhanced CT, flexible endoscopy allows the accurate diagnosis of TIE in more than 90% of cases. The use of endoscopy has been shown to alter surgical management in 69% of patients. In unstable patients rushed to the operative room, intraoperative endoscopy can be employed to rule out esophageal perforation. Under such circumstances triple endoscopy (esophagoscopy, laryngoscopy, and bronchoscopy) is indicated as injury of one of these structures should raise the suspicion of damage to the adjacent organs. Insufflation during the procedure may promote mediastinal contamination by increasing the size of the perforation; for this reason low-flow insufflation and use of CO_2_ rather than air are recommended [104, 113].

#### What are the indications for non-operative management?

Patients with TIE can be offered NOM if they have no esophageal perforation. Patients with esophageal perforation can be offered non-operative management if they meet the previously described NOM criteria (Table 1) (Grade 2A). In these patients, it is mandatory to define the location and the extent of esophageal damage; any delay in the management of overlooked esophageal perforations can impair patient outcomes. It is also essential to detect associated injuries that may affect management and survival [104].

NOM for TIE should be offered only if intense monitoring in an intensive care unit setting, surgical expertise and interventional radiology skills are available around the clock (Grade 1C). NOM requires keeping patients on *nil* per os status, use of broad spectrum antibiotic coverage, endoscopic placement of a nasogastric tube, and early introduction of nutritional support via the use of either enteral feeding or total parenteral nutrition. Additional measures may target the control of sepsis by using percutaneous radiological drainage of peri-esophageal collections, percutaneous chest tube placement and the drainage of pleural collections and pleural decortication by video-thoracoscopy [78, 104–106, 111].

#### What are the indications for immediate surgical treatment?

Patients with TIE should undergo immediate surgical treatment if they have hemodynamic instability, obvious non-contained extravasation of contrast material and systemic signs of severe sepsis (Grade 1C). In these patients, surgery should be undertaken as soon as possible; a large body of literature shows that delayed (> 24 h) surgical management of esophageal perforation results in increased morbidity and mortality rates. Recent studies suggested that while delayed surgical treatment does not affect mortality rates, it did nevertheless reduce the odds of successful primary esophageal repair. If emergency surgery was prompted by associated injuries an esophageal perforation should be sought intraoperatively by direct inspection, intraluminal instillation of dye (methylene blue), or endoscopic insufflation [78, 109, 111].

Delayed surgical treatment is indicated in patients with TIE-related esophageal perforation in whom primary repair of the esophagus was not feasible or had failed (Grade 2A). TIE patients with esophageal perforation who are ineligible for primary repair undergo either esophageal resection or exclusion-diversion procedures. If they survive these, patients require a second procedure to restore continuity of the gastrointestinal tract. Esophageal reconstruction by colon or gastric interposition is usually scheduled 6–12 months after TIE [104].

#### What are the most appropriate surgical procedures?

TIE are rare but highly morbid. Management is dictated by location of the perforation and any concurrent injuries. The majority of cases are amenable to primary repair with flap re-enforcement. Other principles include adequate drainage around the repair, decompression of the esophagus and stomach (via nasogastric tube or gastrostomy tube), and distal enteral nutrition (feeding jejunostomy) [116].

For TIE located in the neck, direct repair of the esophageal perforation should be attempted whenever feasible (Grade 1C). If direct repair is not feasible, esophagostomy and cervical drainage is recommended (Grade 2A). Appropriate treatment of associated injuries (tracheal, carotid) is essential under these circumstances as these can pose specific problems (tracheo-esophageal fistula, postoperative carotid disruption). Avoiding formation of a tracheotomy, buttressing repairs with viable tissue, and drainage through the contralateral neck have all been recommended to prevent such complications [78, 104].

Operative repair is the treatment of choice for TIE with free perforation of the thoracic esophagus (Grade 1C). If primary repair is not feasible, diversion, exclusion, or resection of the thoracic esophagus should be performed (Grade 2A). Severe damage to the spine, the great vessels, the heart, and the lungs may be associated and will determine survival in the short term; their treatment takes priority over esophageal injuries and may require a damage control approach [78, 104].

Operative repair is the treatment of choice for TIE with free perforation of the abdominal esophagus (Grade 1C). Control of potential life-treatment bleeding from associated liver, spleen, or great vessel injuries is essential in patients with abdominal TIE [78, 104].

#### What is the role of damage control surgery?

Principles of damage control surgery and of damage control reanimation should be applied to hemodynamically unstable patients with TIE (Grade 1C). In one study, mortality of TIE was 44% with 92% of the deaths occurring within 24 h of presentation; mortality was related to the injury severity score (ISS) and not to the esophageal injuries [105]. Thus, abbreviated source control surgery followed by transfer to the intensive care unit for physiological resuscitation is paramount in hemodynamically unstable TIE patients; a second look procedure in the operating room is then required for definitive surgical management of esophageal and other associated injuries. External drainage, esophageal exclusion, or expeditious resection should be undertaken in parallel with bleeding control measures; specific treatment of the esophageal lesions would be undertaken in survivors as previously described [111].

## Conclusion

The current recommendations rely on extensive review of the literature and expert opinion. Because of the low incidence of esophageal injuries, high-quality evidence is lacking and the majority of publications in the literature are case reports, case series, or literature reviews. Despite these limitations, the value of the consensus conference in Bertinoro was to gather a panel of recognized experts who discussed point by point all the major issues related to esophageal injuries (Table 2). We recommend a high degree of suspicion in clinical situations that might be associated with or secondarily lead to esophageal perforation; starting appropriate treatment within 24 h can be lifesaving under these circumstances. Both CT and endoscopy are reliable diagnostic tools and their use should be tailored to the patient condition. Definitive management of esophageal emergencies should be undertaken in specialized centers in which multispecialty (esophageal surgeons, interventional radiologists, endoscopists, intensive care unit specialists) expertise is available round the clock.

**Table 2:** Main management principles of esophageal injuries

Foreign body ingestion (FB)	
• Computed tomography (CT) is the key exam in patients with suspected perforation or other FB-related complications	
• Emergent endoscopy (< 6 h) is recommended for sharp-pointed objects, batteries, magnets and for complete esophageal obstruction	
• Indications for surgery include perforation and FB which are irretrievable or close to vital structures	
• Esophagotomy with FB extraction and primary closure is the preferred approach.	
Caustic ingestion	
• The quantity of the ingested agent and the accidental-voluntary ingestion pattern condition outcomes	
• Emergency management can be performed safely relying on computed tomographic evaluation alone	
• Endoscopy remains the main diagnostic and therapeutic tool for caustic strictures	
• Patients who do not have full-thickness necrosis of digestive organs can be offered non-operative management (NOM) under close clinical and biological monitoring. Emergency resection of caustic necrosis can be lifesaving.	
Esophageal perforation (EP)	
• Contrast-enhanced CT and CT esophagography is the imaging examination of choice	
• NOM can be offered to stable patients with early presentation, contained esophageal disruption and minimal contamination of surrounding spaces. Endoscopic (clips, stents) treatment and interventional radiology techniques are useful adjuncts during NOM	
• Emergency surgery should be undertaken in patients who do not meet NOM criteria. Direct repair and adequate drainage is the treatment of choice; if repair is not feasible (large disruption, delayed surgery, preexistent esophageal disease), external drainage, esophageal exclusion or resection are possible options.	
Esophageal trauma	
• Physical examination and laboratory studies are not useful for early diagnosis of TIE.	
• Contrast-enhanced CT and CT esophagography should be performed in hemodynamically stable patients with suspicion of TIE. Preoperative flexible endoscopy is useful for TIE diagnosis in unstable patients	
• Patients with TIE can be offered NOM if they do not have EP or if they meet NOM criteria for EP	
• Patients with TIE should undergo immediate surgical treatment if they have hemodynamic instability, obvious non-contained extravasation of contrast material and systemic signs of severe sepsis	
• Operative repair is the treatment of choice of TIE. Appropriate management of associate injuries conditions patient survival	

## Data Availability

Not applicable

## Abbreviations

CBC: Complete blood count
CRP: C-reactive protein
EI: Esophageal injuries
EP: Esophageal perforation
FB: Foreign bodies
NOM: Non-operative management
TIE: Traumatic injuries of the esophagus
